# Constructing ordinal partition transition networks from multivariate time series

**DOI:** 10.1038/s41598-017-08245-x

**Published:** 2017-08-10

**Authors:** Jiayang Zhang, Jie Zhou, Ming Tang, Heng Guo, Michael Small, Yong Zou

**Affiliations:** 1 Department of Physics, East China Normal University, Shanghai, 200241 China; 2 School of Information Science Technology, East China Normal University, Shanghai, 200241 China; 3 School of Mathematics and Statistics, University of Western Australia, Crawley, WA 6009 Australia; 4 Mineral Resources, CSIRO, Kensington, WA Australia

## Abstract

A growing number of algorithms have been proposed to map a scalar time series into ordinal partition transition networks. However, most observable phenomena in the empirical sciences are of a multivariate nature. We construct ordinal partition transition networks for multivariate time series. This approach yields weighted directed networks representing the pattern transition properties of time series in velocity space, which hence provides dynamic insights of the underling system. Furthermore, we propose a measure of entropy to characterize ordinal partition transition dynamics, which is sensitive to capturing the possible local geometric changes of phase space trajectories. We demonstrate the applicability of pattern transition networks to capture phase coherence to non-coherence transitions, and to characterize paths to phase synchronizations. Therefore, we conclude that the ordinal partition transition network approach provides complementary insight to the traditional symbolic analysis of nonlinear multivariate time series.

## Introduction

Nonlinear time series analysis and complex network theory are widely considered to be established fields of complex systems sciences with strong links to nonlinear dynamics and statistical physics. There has been a growing body of literature aimed at the utilization of complex network methods for characterizing dynamical systems based on time series. There are various ways to transforming a given time series to a network representation and then to do network analysis. Here we give a few typical examples. Recurrence network approaches compare the closeness of time points in phase space, which have been applied to climate data analysis^1, 2^, chaotic electro-chemical oscillators^3^, fractional Brownian motion^4^, and oil-water two phase transitional flow behavior^5–7^. Some basic network motif structures have been identified in musical data^8^, which has been further characterized by revised recurrence approaches^9^. A series of visibility graph algorithms and their variants^10–12^ have been proposed to transform a given time series by computing a so-called linear visibility condition between each pair of two sampled points, which have been successfully applied to hurricane data in the US^13^, financial market^14^, sunspot time series^15, 16^, correlated stochastic^17^ and multi-fractal stochastic processes^18^, providing novel insights from a complex systems perspective. Several other methods have been discussed in refs 8, 19 and 20. For instance, the idea of cycle network is proposed for mapping a time series to a network^21^. Characterizing the order of motifs is helpful to distinguish high-dimensional chaos from low-dimensional chaos^22^. In addition, one can monitor the evolutionary behavior of a time series by mapping segments of a time series to a visibility graph and linking the successive states to a state network^23, 24^.

Recently, there is a growing number of works in transforming time series into networks by ordinal partitions of time series^25, 26^. A series of systematic investigations of ordinal methods has been conducted in irregularly sampled time series^27–29^, which shows high potential for studies of experimental observation data from climate sciences^30^. In this method, the first step is to embed a one-dimensional time series into phase space by using techniques from traditional time delay embedding. Then, embedded points in phase space are mapped to nodes in the network space and links are allocated between nodes based on temporal succession on the trajectory. The resulting network is a Markov chain representation of the time series in phase space. The interesting point of network analysis is that rather simple network measures including even mean degrees can track the dynamical transitions comparable to the largest Lyapunov exponent^25^.

The basic idea of ordinal partition network method can be traced back to identifying ordinal patterns of time series^31, 32^. Considering a one-dimensional time series ${\{x(t)\}}_{t=1,\ldots ,L}$ comprising of *L* points from a dynamical system, the original phase space can be reconstructed by time delay embedding $\overrightarrow{x}(t)=[x(t),x(t+\tau ),\ldots ,$ $x(t+({D}_{x}-\mathrm{1)}\tau )]$ with dimension *D*_*x*_^33, 34^. The next step is to compute the rank order of $x(t),x(t+\tau ),\ldots ,$ $x(t+({D}_{x}-\mathrm{1)}\tau )$ based on relative amplitudes, which is conveniently represented by a symbol *π*_*x*_(*t*). When sliding windows from *t* = 1 to *N* = *L* − (*D*_*x*_ − 1)*τ* in the embedded space, a symbolic representation of the trajectory *π*_*x*_(*t*) is produced. One traditional approach, following the symbolic representations, is to compute permutation entropy based on the frequency plot of order pattern which yields very well established statistical measures in nonlinear time series analysis^31^. In the recent decades, ordinal symbolic representation of time series has found a number of interesting applications in science and engineering, for instance, biomedical recordings^35^, finance^36^, climate sciences^37^. Some recent progress has been comprehensively reviewed in ref. 35. However, the transition behavior between ordinal patterns remains largely unclear. The recent ordinal partition network representations capture the evolutionary behavior of the ordinal patterns^25, 26^, which sheds novel insight on the standard ordinal symbolic analysis of time series.

For a given embedding dimension *D*_*x*_, there are a total of *D*_*x*_! unique ordinal patterns that can possibly occur in a time series, neglecting equality. A stochastic process of stationary increments fulfils *P*(*x*_*t*_ = *x*_*t*+*τ*_) = 0 and therefore the probability to have ties *x*_*t*_ = *x*_*t*+*τ*_ is zero. For empirical time series, we can avoid ties by adding a tiny white noise with continuous distribution^38^. Therefore, the original phase space is decomposed into *D*_*x*_! equivalent partitions^31^. It is intuitive that all *D*_*x*_! patterns almost occur with equal frequencies in a time series generated by a stochastic process for *N* → ∞. However, a set of patterns may never occur in a time series produced by deterministic dynamics. Therefore, it is possible to quantify determinism in time series data by counting the forbidden patterns. However, complications arise in real time analysis. For instance, missing ordinal patterns might be related to finite time length during the period of observation and correlated stochastic processes, which require some revised methods for the detection of determinism in relatively short noisy data^39–43^. From the viewpoint of ordinal partition networks, both the frequencies of order patterns and the transitions between different patterns are inhomogeneous. Therefore, network properties thus obtained are sensitive to different system dynamics, which successfully characterize the difference between healthy and patients from EEG data^25, 26^.

Most of the recent works have focused only on univariate time series {*x*(*t*)}. The embedding dimension *D*_*x*_ and time delay *τ* are two important parameters for constructing ordinal partition networks, in particular having crucial impacts on the appearance of forbidden order patterns^27–29^. However, the generalization to multivariate time series remains largely untouched^5, 44^. Most of the observable phenomena in the empirical sciences are of a multivariate nature. For instance, assets in stock markets are observed simultaneously and their joint development is analyzed to better understand tendencies. In climate science, multiple observations (temperature, pressure, precipitation, human activities etc, from different locations) are the basis of reliable predictions for the future climate conditions. We propose to construct ordinal partition transition networks from multivariate data.

## Results

### Ordinal pattern definitions

Given a scalar time series {*x*(*t*)} which is produced by a deterministic dynamical system, the order structure of the time series depends on the embedding dimension *D*_*x*_ and time delay *τ* ^33, 34^. Let us start with *D*_*x*_ = 2. Neglecting equality, we have two relations between *x*(*t*) and *x*(*t* + *τ*), namely, two symbol sequences representing order patterns *π*_*x*_:

1 $${\pi }_{x}(t)=\{\begin{array}{cc}1 & {\rm{i}}{\rm{f}}\,x(t) < x(t+\tau ),\\ 0 & {\rm{i}}{\rm{f}}\,x(t) > x(t+\tau ).\end{array}$$

For dynamical systems with continuous distributions of the values, we can neglect equality because the Lebesgue measure of ties is zero^31^. In addition, a large amount of numerical simulations suggest that the results do not change qualitatively and it does not matter whether we count *x*(*t*) < *x*(*t* + *τ*) or *x*(*t*) ≤ *x*(*t* + *τ*)^38, 45^. In the practical application, we may easily test for < and ≤. Therefore, we follow the routine as suggested in ref. 45 and do not separately consider equalities. Time delay *τ* is chosen as 1 in this work. By this choice, the order pattern ${\pi }_{x}^{1}$ captures the increasing trend, respectively, ${\pi }_{x}^{0}$ corresponds to the decreasing trend of the time series. This definition is equivalent to considering the signs of the increments Δ*x*(*t*) = *x*(*t* + 1) − *x*(*t*) by a first-order difference of the original series. In other words, the associated order patterns capture the variations of *x*(*t*) in its velocity space, showing dynamic rather than static information based on the displacement directly.

**Figure 1:**
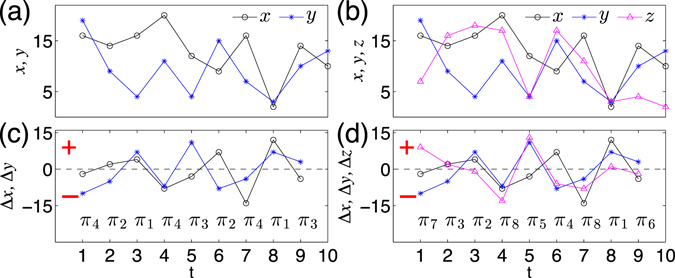
Order pattern definitions for (**a**) two dimensional series (*x*(*t*), *y*(*t*)) and (**c**) its increment series (Δ*x*(*t*), Δ*y*(*t*)). (**b**) Three dimensional series (*x*(*t*), *y*(*t*), *z*(*t*)) and (**d**) the corresponding increment series (Δ*x*(*t*), Δ*y*(*t*), Δ*y*(*t*)). Signs of the increment series and the ordinal patterns are respectively indicated in (**c**,**d**).

When generalizing the above idea to two dimensional time series (*x*(*t*), *y*(*t*)), we restrict our discussion on embedding dimension *D*_*x*_ = *D*_*y*_ = 2 for individual variable. Therefore, we have four different combinations of order patterns depending on the signs of increments (Δ*x*(*t*), Δ*y*(*t*)) (Table 1): In the phase space of (*x*(*t*), *y*(*t*)), we have order pattern Π(*t*) ∈ (*π*_1_, *π*_2_, *π*_3_, *π*_4_) which captures the increasing or decreasing behavior. The example of Fig. 1(a,c) shows the construction of ordinal patterns for two dimensional series (*x*(*t*), *y*(*t*)). In a full analogy, based on increments (Δ*x*(*t*), Δ*y*(*t*), Δ*z*(*t*)), ordinal pattern ${\rm{\Pi }}(t)\in ({\pi }_{1},\ldots ,{\pi }_{i}),\,i=1,\ldots \mathrm{,\; 8}$ of a three dimensional time series (*x*(*t*), *y*(*t*), *z*(*t*)) is enumerated in Table 2 and visualized in Fig. 1(b,d). Therefore, the dimension of order pattern Π(*t*) for an *n*-dimensional time series $(\{{x}_{1}\}(t),\ldots ,\{{x}_{n}\}(t))$ is *D* = 2^*n*^ since each component has either increasing or decreasing trend at time *t*.

**Table 1:** Order patterns in two dimensional time series (*x*(*t*), *y*(*t*)).

Π	*π*_1_	*π*_2_	*π*_3_	*π*_4_
*X*	${\pi }_{x}^{1},+$	${\pi }_{x}^{1},+$	${\pi }_{x}^{0},-$	${\pi }_{x}^{0},-$
*Y*	${\pi }_{y}^{1},+$	${\pi }_{y}^{0},-$	${\pi }_{y}^{1},+$	${\pi }_{y}^{0},-$

**Table 2:** Order patterns in three dimensional time series (*x*(*t*), *y*(*t*), *z*(*t*)).

Π	*π*_1_	*π*_2_	*π*_3_	*π*_4_	*π*_5_	*π*_6_	*π*_7_	*π*_8_
*X*	${\pi }_{x}^{1},+$	${\pi }_{x}^{1},+$	${\pi }_{x}^{1},+$	${\pi }_{x}^{1},+$	${\pi }_{x}^{0},-$	${\pi }_{x}^{0},-$	${\pi }_{x}^{0},-$	${\pi }_{x}^{0},-$
*Y*	${\pi }_{y}^{1},+$	${\pi }_{y}^{1},+$	${\pi }_{y}^{0},-$	${\pi }_{y}^{0},-$	${\pi }_{y}^{1},+$	${\pi }_{y}^{1},+$	${\pi }_{y}^{0},-$	${\pi }_{y}^{0},-$
*Z*	${\pi }_{z}^{1},+$	${\pi }_{z}^{0},-$	${\pi }_{z}^{1},+$	${\pi }_{z}^{0},-$	${\pi }_{z}^{1},+$	${\pi }_{z}^{0},-$	${\pi }_{z}^{1},+$	${\pi }_{z}^{0},-$

Note that, in this work, we do not apply time delay embedding technique to obtain the multi-dimensional phase space from one univariate time series. Instead, given multi-variate time series, we consider the increments between two consecutive time points of each measurement in the space of multi measurements. In other words, our approach captures the dynamic properties of the multi-variate time series in its associated velocity space (difference space). Therefore, time delay *τ* in the order pattern definition (Eq. (1)) has rather a different interpretation with the time delay that is often used in embedding. Traditionally, one chooses appropriately an embedding dimension and time delay to reconstruct phase space from a given univariate time series. We can certainly generalize the discussion to the case of time delays larger than 1 (i.e., *τ* > 1) and embedding dimension *D*_*x*_ > 2 for each variable (measurement), but we think that the physical meaning in terms of dynamics becomes ambiguous for multivariate time series.

### Ordinal partition transition networks

Given a multi-variate time series, for instance, the two dimensional case of (*x*(*t*), *y*(*t*)), we denote the frequency of the *i*-th pattern *π*_*i*_ as *p*(*π*_*i*_) which is computed over the time interval $t=1,\ldots ,N$. One important property is that ordinal patterns from a deterministic process have different frequencies *p*(*π*_*i*_). Permutation entropy ${{\mathcal{H}}}_{O}$ is then introduced to characterize the inhomogeneous appearance of ordinal patterns as following

2 $${ {\mathcal H} }_{O}=-\sum _{i=1}^{{2}^{n}}\,p({\pi }_{i})\,{\mathrm{log}}_{2}\,p({\pi }_{i}),$$

where the sum runs over all *D* = 2^*n*^ permutations. We use log_2_ and hence the units of ${ {\mathcal H} }_{O}$ are bits. For a *n*-dimensional independent identical distributed stochastic process, one obtains the largest entropy ${ {\mathcal H} }_{O}=n$ since each of *D* = 2^*n*^ ordinal patterns is expected to have the same frequency.

**Figure 2:**
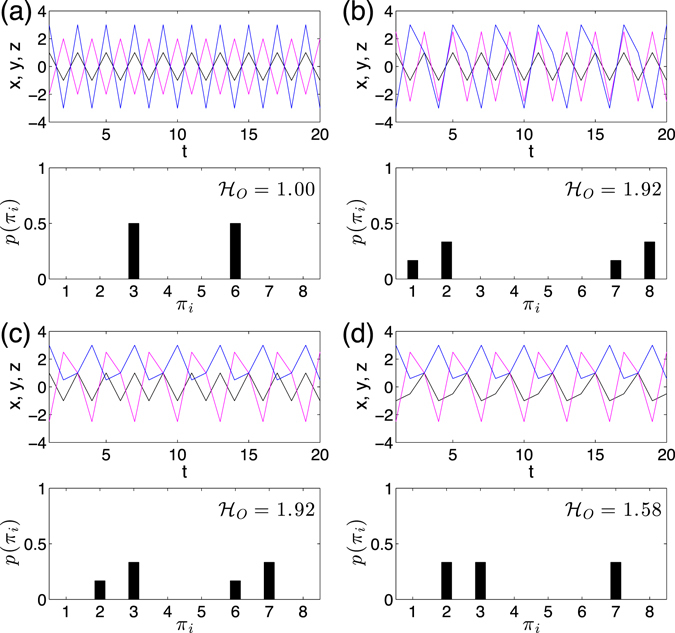
A toy model of periodic 3D series (*x*(*t*), *y*(*t*), *z*(*t*)) and its associated histogram of order patterns. (**a**) *x*(*t*), *y*(*t*), and *z*(*t*) has the same period 2. (**b**) *x*(*t*) and *y*(*t*) have period 2, but *z*(*t*) has period 3. (**c**) *x*(*t*) has period 2, *y*(*t*) and *z*(*t*) have period 3. (**d**) *x*(*t*), *y*(*t*) and *z*(*t*) have the same period 3. The respective frequency plot of the ordinal patterns is shown below the time series and entropy values ${ {\mathcal H} }_{O}$ are indicated in the legends.

We illustrate the above algorithm by using a toy model of three dimensional identical independent periodic time series in Fig. 2. Different combinations of periods of the 3D periodic series often yield different values of permutation entropy ${ {\mathcal H} }_{O}$. In addition, the limited number of possible ordinal patterns (forbidden patterns) are widely observed reflecting the determinism of the series.

**Figure 3:**
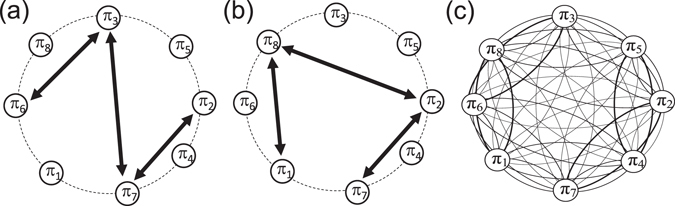
(**a**) Ordinal partition transition networks for periodic processes of Fig. 2(b), and respectively, (**b**) is for Fig. 2(c). Panel (c) corresponds to a 3D independent identical distributed random uniform noise where link arrows (bidirectional) are suppressed for the ease of visualization.

Most of the current studies focused on the computation of permutation entropy ${ {\mathcal H} }_{O}$ considering the frequencies of order patterns, which do *not* disclose the transition behavior between order patterns. Therefore, ${ {\mathcal H} }_{O}$ is static. For instance, the details of the dynamics remain unclear in Fig. 2(b,c) provided only with the values of ${ {\mathcal H} }_{O}=1.92$, because ${ {\mathcal H} }_{O}$ does not disclose the unique transition properties of Fig. 2(b) from (c). From the viewpoint of visualization, the difference of transitions between order patterns are conveniently shown in Fig. 3(a,b). Comparing to a 3-dimensional uncorrelated independent identical distributed random uniform noise, the ordinal partition network is a complete connected graph (Fig. 3(c)). In addition, we indicate each directed link by its transition frequency *w*_*ij*_ = *p*(*π*_*i*_ → *π*_*j*_), following the time iterations of the series. Finally, we come up with a weighted directed network characterized by a weighted adjacency matrix *W* = {*w*_*ij*_}, *i*, *j* ∈ [1, 2^*n*^]. The matrix *W* fulfils the normalization ${\sum }_{i,j}^{{2}^{n}}\,{w}_{ij}=1$. Here, based on *W*, the regularity of the order pattern transition properties is quantified by the Shannon entropy ${ {\mathcal H} }_{T}$, which is

3 $${ {\mathcal H} }_{T}=-\sum _{i,j=1}^{{2}^{n}}\,{w}_{ij}\,{\mathrm{log}}_{2}\,{w}_{ij},$$

where the sum runs over all possible 2^2*n*^ transitions. In a full analogy to ${ {\mathcal H} }_{O}$, for a *n*-dimensional independent identical distributed stochastic process, one obtains the largest entropy ${ {\mathcal H} }_{T}=2n$.

**Figure 4:**
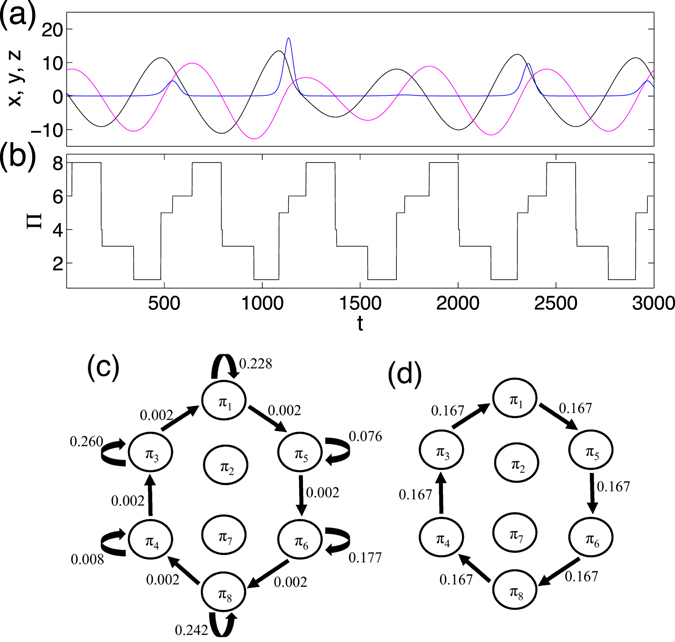
Chaotic Rössler system (*a* = 0.165): (**a**) short segments of time series (*x*, *y*, *z*), (**b**) temporal variation of order patterns corresponding to the particular time window of (**a**,**c**) ordinal pattern transition network with self-loops, ${ {\mathcal H} }_{O}=2.283$, and (**d**) without self-loops, ${ {\mathcal H} }_{T}=2.585$, where the transition route *π*_1_ → *π*_5_ → *π*_6_ → *π*_8_ → *π*_4_ → *π*_3_ → *π*_1_ is observed. The values on links represent the corresponding transition frequencies of the ordinal patterns. Note that *N* = 500000 data points are used in obtaining (**c**,**d**).

It is rather straightforward to compute ${ {\mathcal H} }_{T}$ for time series produced by stochastic processes. However, we need to pay attention to the case of continuous systems, which yield a larger proportion of self-loops in the resulted networks as will be illustrated below. Here we take the chaotic Rössler system as an example which reads

4 $$\begin{array}{ccl}\dot{x} & = & -\omega y-z,\\ \dot{y} & = & \omega x+ay,\\ \dot{z} & = & 0.4+z(x-\mathrm{8.5),}\end{array}$$

where *a* = 0.165, and *ω* = 1.0. The Eq. (4) are numerically integrated by the fourth-order Runge Kutta method with integration step *h* = 0.01. The first 10000 transient data points are discarded and time series consisting of *N* = 500000 data points are analyzed. Short segments of time series (*x*, *y*, *z*) are shown in Fig. 4(a). Due to the continuity property of the system, there are many plateaus reflecting the invariance of the order patterns during certain time intervals (Fig. 4(b)). These plateaus are reflected by self-loops in the resulting transition networks (Fig. 4(c)). In most of the existing studies of complex networks, self-loops are avoided because of both the computational simplicity and theoretical concerns^46^. In the case of the Rössler system, there is about 99% self-loops and only about 1% non-self-loops (as indicated by the arrows in Fig. 4(c)).

The weighted adjacency matrix *W* can be split into diagonal and off-diagonal entries. Taking into account the numerical observations that the diagonal elements of *W* (self-loops) are much larger than the off-diagonal ones, Eq. (3) is simplified to

5 $$\begin{array}{rcl}{ {\mathcal H} }_{T} & = & -\sum _{i,j=1,i\ne j}^{{2}^{n}}\,{w}_{ij}\,{\mathrm{log}}_{2}\,{w}_{ij}-\sum _{i,j=1,i=j}^{{2}^{n}}\,{w}_{ij}\,{\mathrm{log}}_{2}\,{w}_{ij},\\  & \approx  & -\sum _{i=1}^{{2}^{n}}\,{w}_{ii}\,{\mathrm{log}}_{2}\,{w}_{ii}\\  & = & -\sum _{i=1}^{{2}^{n}}\,p({\pi }_{i})\,{\mathrm{log}}_{2}\,p({\pi }_{i}).\end{array}$$

Therefore, we obtain ${ {\mathcal H} }_{T}\approx { {\mathcal H} }_{O}$ for a continuous system when self-loops are considered. In this case, the transitions between different ordinal patterns are hard to be captured by ${ {\mathcal H} }_{T}$.

In order to emphasize the importance of non-self transitions between ordinal patterns, we remove the self-loops as shown in Fig. 4(d) by setting the diagonal values to be 0 in *W*. This is typical of most research work on complex networks^46^. Furthermore, we remove self-loops before computing the weighted matrix *W* to keep the normalization ${\sum }_{i,j}^{{2}^{n}}\,{w}_{ij}=1$. Note that self-loops should not be expected with large amounts in stochastic processes.

### Ordinal pattern partitions of phase space

Ordinal pattern transition networks provide us with an alternative for phase space partitions, which utilizes nullclines of the systems. Here we show two examples covering discrete and continuous dynamical systems.

**Figure 5:**
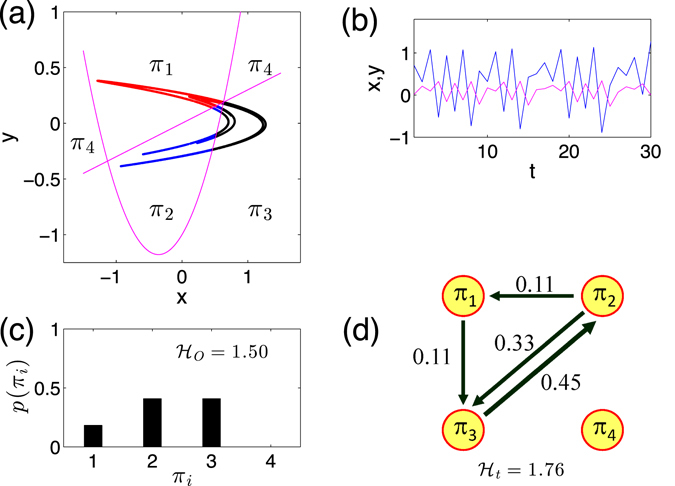
Hénon map: (**a**) attractor, (**b**) segments of time series, (**c**) histogram of order patterns leads to ${ {\mathcal H} }_{O}=1.5$, and (**d**) ordinal pattern transition network removing self-loops which yields ${ {\mathcal H} }_{T}=1.76$.

*Example* (*1*): *the Hénon map*

6 $$\begin{array}{rcl}x(t+\mathrm{1)} & = & y(t)+1-1.4{x}^{2}(t),\\ y(t+\mathrm{1)} & = & 0.3x(t),\end{array}$$

is chosen as an example for a chaotic two-dimensional map. The order pattern partitions of the attractor are shown Fig. 5(a), which is color coded by different order patterns. A segment of time series is shown in Fig. 5(b). The histogram of order patterns (Fig. 5(c)) discovers that *π*_4_ are forbidden patterns of the system, which yields ${ {\mathcal H} }_{O}=1.50$. The corresponding ordinal partition transition network is shown in Fig. 5(d) and the frequency of each link is indicated in the figure, which yields ${ {\mathcal H} }_{T}=1.76$.

According to the order pattern definitions in Table 1, the phase space partitions of the Hénon map are delineated by nullclines as follows:

7 $$\begin{array}{lll}{L}_{1} & : & y(t)+1-1.4{x}^{2}(t)-x(t)=\mathrm{0,}\\ {L}_{2} & : & 0.3x(t)-y(t)=0.\end{array}$$

These two lines are shown in Fig. 5(a), where we find no points of the attractor lying in the region of order pattern *π*_4_ no matter with the iteration steps. The disappearance of *π*_4_ pattern suggests that there is no intersection between the *π*_4_ partition and the attractor, except for the unstable fixed point of (0.63, 0.19) (intersection of *L*_1_ and *L*_2_).

**Figure 6:**
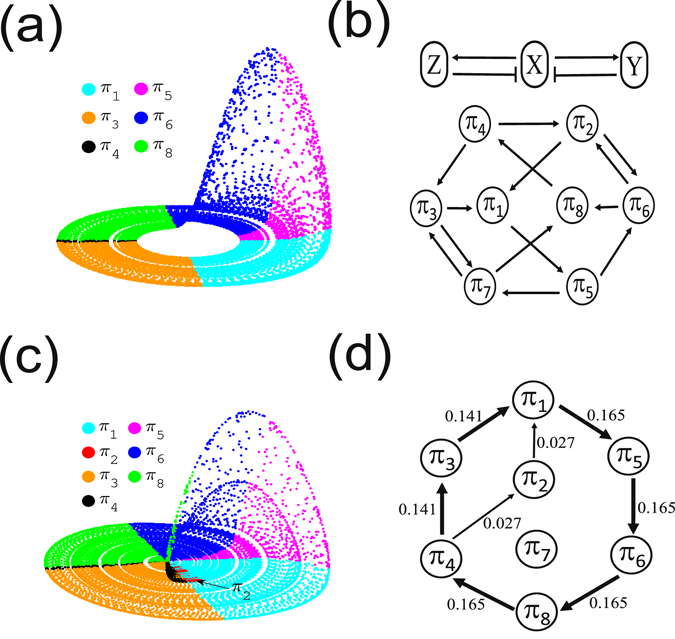
(**a**) Rössler attractor (*a* = 0.165) in phase space color coded by ordinal patterns, which are indicated by legends. Patterns *π*_2_ and *π*_7_ are not observed. (**b**) Upper panel is the activation-repression relationship between variables *x*, *y* and *z*, where activation is denoted by a normal arrow, and repression by a barred arrow^47^; lower panel represents all allowed (not necessarily observed) pattern transitions of the system. The corresponding ordinal partition transition network is shown in Fig. 4(d). Panel (c) is the same as (**a**) with *a* = 0.26, where a significant number of *π*_2_ patterns are highlighted, and (**d**) is the ordinal partition transition network (self-loops are excluded), where an alternative transition from *π*_4_ → *π*_2_ → *π*_1_ has been observed.

*Example* (*2*): *the Rössler system*, Eq. 4 is chosen as a continuous dynamical system. When the parameter *a* = 0.165, the attractor is shown in Fig. 6(a) with phase space points being further color-coded by ordinal patterns. The boundaries of each partition are determined by the corresponding nullclines, i.e., *dx*/*dt* = 0, *dy*/*dt* = 0 and *dz*/*dt* = 0. The ordinal pattern transition network is shown in Fig. 4(d). In this case, neither *π*_2_ nor *π*_7_ appears, which is explained as follows^47^. When transforming phase space into ordinal partition transition network, we associate to each state (*x*, *y*, *z*) with an order pattern such as (+, −, +) (as listed in Table 2). This 3-dimensional ordinal pattern describes which variables of (*x*, *y*, *z*) are increasing and which are decreasing at a given time. Taking variable *x* as an example, both *y* and *z* are repressors to *x* because of the negative signs in the Jacobian (−*ω*, and −1). However, *x* is an activator for variable *y* because the element of the Jacobian matrix is *ω* being positive everywhere in phase space. The scheme of activation and repression of the Rössler system is shown in Fig. 6(b).

The condition that the trajectory can cross a nullcline meaning a change from increasing to decreasing (or vice versa) are equivalent to determining local maximum or minimum. Because of the continuity of the system, we have the following rules to have a maximum or minimum^47^: (i) a variable cannot have a maximum if all its repressors are decreasing and all its activators are increasing; (ii) a variable cannot have a minimum if all its repressors are increasing and all its activators are decreasing. These two rules yield all possible transitions between different order patterns as shown in Fig. 6(b). However, the transition network observed for a typical trajectory in phase space is determined by the given set of parameters *a* and *ω*. For the case *a* = 0.165 and *ω* = 1, we only find the transition route *π*_1_ → *π*_5_ → *π*_6_ → *π*_8_ → *π*_4_ → *π*_3_ → *π*_1_ (shown in Fig. 4(d)), and meanwhile *π*_2_ and *π*_7_ are forbidden patterns. Increasing the value *a* to 0.26, the Rössler system presents screw-type chaotic oscillations with irregular kicks, which yield an alternative transition from *π*_4_ → *π*_2_ → *π*_1_ as highlighted in Fig. 6(c). Therefore, we observe two transition routes of the patterns from *π*_4_ to *π*_1_ (Fig. 6(d)), while *π*_7_ remains to be absent. In other words, the appearance of *π*_2_ pattern suggests that the changes of the ordinal patterns are sensitive to the geometric changes of the attractor.

### Identifying dynamical transitions

We apply ordinal partition transition networks to identify dynamical transitions in two different cases: (i) phase coherence to non-coherence transition which is a weak chaos-chaos transition, (ii) paths to phase synchronization transitions. In both examples, we show frequency plots of ordinal patterns (without self-loops), complexity entropy measures of ${ {\mathcal H} }_{O}$ (with self-loops) and ${ {\mathcal H} }_{T}$ (without self-loops). In addition, we compare the case (i) to coherence index (CI), and case (ii) to mean rotation frequency of each oscillator Ω_*i*_.

*Example* (*1*) *shows phase coherence to non*-*coherence transitions* in the chaotic Rössler system (Eq. (4)), where the parameter *a* is systematically varied in the range [0.15, 0.25]. As it has been systematically shown in ref. 48, this parameter range comprises different kinds of dynamics, including periodic windows, phase coherent chaos (existence of a well-defined rotation center in phase space) as well as non-phase coherent chaotic oscillations (lack of a distinct center of rotation). The transition between phase coherence and non-phase coherence chaos occurs at *a*_*c*_ ≈ 0.206. More specifically, for *a* < *a*_*c*_, the chaotic attractors are always phase coherent, whereas they are non-phase coherent for *a* > *a*_*c*_. We refer readers to ref. 48 for further discussion on various measures to detect this chaos-chaos transition as well as periodic windows, ranging from traditional measures of phase coherence factor, phase diffusion coefficient, recurrence quantification based discriminators, and recurrence network based measures. In this work, in order to avoid repetitions we only discuss the capabilities of ordinal pattern changes and entropies ${ {\mathcal H} }_{O}$ and ${ {\mathcal H} }_{T}$ in detecting the transition from phase coherent to non-coherent chaos by comparing to the measure of phase coherence index (see Methods).

**Figure 7:**
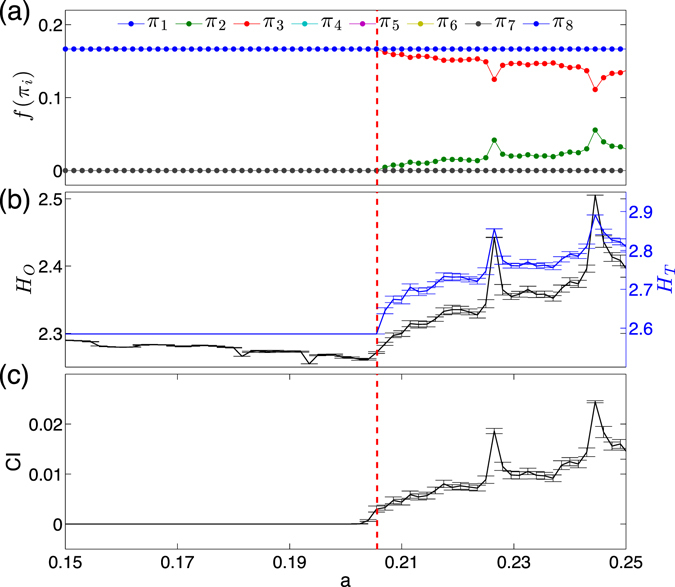
Phase coherence to non-phase coherence transition for the Rössler system as a function of the parameter *a* (error bars indicate standard deviations obtained from 100 independent realizations of the system for each value of *a*: (**a**) frequency of each ordinal pattern *f*(*π*_*i*_), where *π*_1_, *π*_4_, *π*_5_, *π*_6_ and *π*_8_ are overlapped in the entire range of *a*. (**b**) Entropy values ${ {\mathcal H} }_{O}$ and ${ {\mathcal H} }_{T}$, (**c**) coherence index (CI). The transition from phase coherent to non-coherent is highlighted by the vertical dashed lines.

Figure 7 shows the bifurcation diagram when the parameter *a* is changed. First, the frequency of ordinal pattern *π*_2_ is zero (*f*(*π*_2_) = 0) when *a* < *a*_*c*_, and becomes positive when *a* > *a*_*c*_. In contrast, *f*(*π*_3_) decreases when *a* > *a*_*c*_ (Fig. 7(a)). Much smaller changes are observed for the other ordinal patterns *π*_1_, *π*_4_, *π*_5_, *π*_6_ and *π*_8_. Pattern *π*_7_ does not appear in the entire interval of *a*. Ordinal patterns *π*_2_ and *π*_3_ are sensitive to the geometric changes of the attractor, capturing the transition from phase coherent to non-coherent regime.

In addition, ${ {\mathcal H} }_{O}$ shows rather small changes while ${ {\mathcal H} }_{T}$ is a constant value when *a* < *a*_*c*_ (Fig. 7(b)). The behavior of ${ {\mathcal H} }_{O}$ and ${ {\mathcal H} }_{T}$ has been confirmed by the coherence index (see Methods) as shown in Fig. 7(c). Meanwhile, we find some discrepancies for these measures when the control parameter *a* increases towards the transition point between phase coherence and non-coherence regimes. In particular, both ${ {\mathcal H} }_{O}$ and the coherence index increase slightly before the transition point *a*_*c*_, while ${ {\mathcal H} }_{T}$ increases sharp at *a*_*c*_, as highlighted by vertical dashed lines in Fig. 7. This is because of the homo-clinic point at the origin. As the control parameter *a* increases within the phase coherent regime, the attractor successively grows and finally extends to the vicinity of the origin shortly before the transition to the funnel regime, where a unique rotation center of trajectories in phase space is lost. The dynamics in the (*x*, *y*)-plane becomes very slow whenever a trajectory gets close to the homo-clinic point. As a consequence, there is a high density of sampled points on a trajectory in the neighborhood of the origin. By the same time, these re-injection to and ejection from the origin are rather irregular events, which introduce fluctuations in the computations of ${ {\mathcal H} }_{O}$ and the coherence index. In contrast, when computing ${ {\mathcal H} }_{T}$, the local velocities change direction from increasing to decreasing (or vise versa) only after the transition to the non-phase coherent regime. Therefore, ${ {\mathcal H} }_{T}$ shows good sensitivity on the change of the local velocity space when the control parameter *a* passes the transition from phase coherent to non-coherent regime, which are shown in Fig. 7.

Note that all measures of ${ {\mathcal H} }_{O}$, ${ {\mathcal H} }_{T}$ and coherence index show pronounced local maxima in periodic windows (for instance, at *a* = 0.227 and *a* = 0.245)^48^.

*Example* (*2*) *shows paths to phase synchronization*, which are demonstrated by three diffusively coupled Rössler systems via *x* component^49^. The equations read

8 $$(\begin{array}{cc} & {\dot{x}}_{k}\\  & {\dot{y}}_{k}\\  & {\dot{z}}_{k}\end{array})=(\begin{array}{l}-{\omega }_{k}\,{y}_{k}-{z}_{k}+\sum _{l\ne k}{\kappa }_{k,l}({x}_{l}-{x}_{k})\\ {\omega }_{k}\,{x}_{k}+a{y}_{k}\\ 0.4+{z}_{k}({x}_{k}-8.5)\end{array}),$$

where *k* = 1, 2, 3 and *κ* is the coupling strength. We consider non-identical oscillators by choosing *ω*_1_ = 0.98, *ω*_2_ = 1.02, *ω*_3_ = 1.06. The parameter *a* is chosen as 0.165 such that the subsystems are in the phase coherent regime (Fig. 6(a)). The oscillator *k* = 2 is bidirectionally coupled to both *k* = 1 and *k* = 3, whereas there is no direct coupling between *k* = 1 and *k* = 3. The Eq. (8) are numerically integrated by the fourth-order Runge Kutta method with integration step *h* = 0.01. The first 10000 transient data points are discarded and time series consisting of 150000 data points are analyzed. We construct ordinal pattern transition networks from the *x*_*k*_ components, namely, (*x*_1_, *x*_2_, *x*_3_), following the same pattern definitions as shown in Table 2.

**Figure 8:**
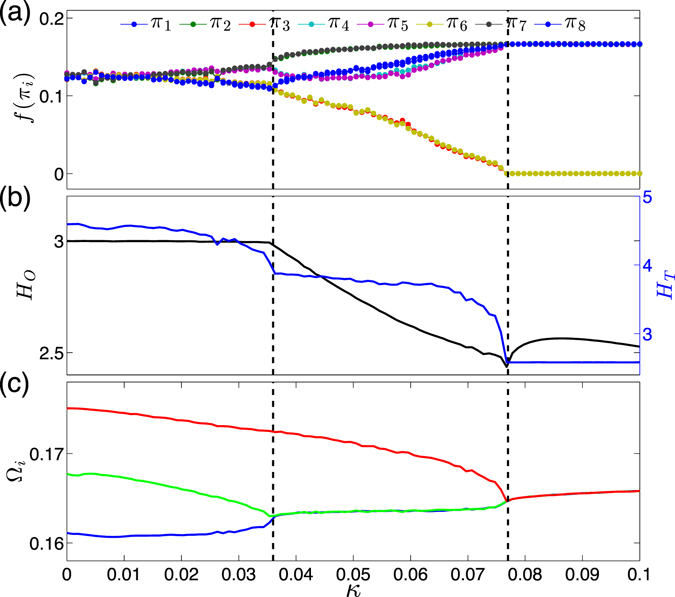
Phase synchronization transitions of three coupled Rössler systems. (**a**) Frequency of each ordinal pattern *f*(*π*_*i*_), (**b**) entropy values ${ {\mathcal H} }_{O}$ and ${ {\mathcal H} }_{T}$, (**c**) mean rotation frequency Ω_*i*_ of each oscillator. Subsystem *k*_1_ and *k*_2_ are synchronized at *κ*_*c*1_ = 0.036, and *k*_3_ joins the synchronization only at a stronger coupling strength *κ*_*c*2_ = 0.077. Both critical coupling values are highlighted by vertical dashed lines.

Our motivation of using Eq. (8) is to study the variations of ordinal patterns on the paths to phase synchronization, focusing on the evolutionary process of the transition networks with different regimes of synchronization, which is more complex than the case of a single Rössler system. The results are shown in Fig. 8. Furthermore, the results of Fig. 8 have been averaged over 50 random initial conditions when integrating Eq. (8).

In the regime of no synchrony (*κ* < *κ*_*c*1_ = 0.036), three oscillators evolve almost independently such that all ordinal patterns have the same frequencies of 0.125. There are rather small gradual changes only when *κ* approaches to *κ*_*c*1_ (Fig. 8(a)). The entropy value ${ {\mathcal H} }_{T}$ is more sensitive to these gradual changes showing a pronounced decreasing trend, while ${ {\mathcal H} }_{O}$ seems to be a constant (Fig. 8(b)). The average rotation frequencies Ω_*k*_ (see Methods) of each oscillator are shown in (Fig. 8(c)), which confirms no synchrony in this coupling regime.

In the regime that phase synchronization appears between oscillators *k* = 1 and *k* = 2, but not with *k* = 3 ($\kappa \in [{\kappa }_{c1},{\kappa }_{c2}]=[0.036,0.077]$), we observe monotonic increasing trends for order patterns *π*_1_, *π*_2_, *π*_7_, and *π*_8_ (Fig. 8(a)). In addition, we find relatively slower increasing trends for patterns of *π*_4_ and *π*_5_. In contrast, some monotonic decreasing trends are found for *π*_3_ and *π*_6_. The changes in the frequencies of order patterns are captured by both entropy values ${ {\mathcal H} }_{O}$ and ${ {\mathcal H} }_{T}$, showing gradual decreasing trends (Fig. 8(b)). The average rotation frequencies Ω_*k*_ are shown in Fig. 8(c), where *k* = 1 and *k* = 2 are phase locked to the same rotation frequency but not with *k* = 3.

In the regime with all oscillators in phase synchronization (*κ* > *κ*_*c*2_ = 0.077), we find that frequencies of patterns *π*_1_, *π*_2_, *π*_4_, *π*_5_, *π*_7_, *π*_8_ converge to the same value *f*(*π*_*i*_) = 1/6, while *π*_3_ and *π*_6_ are absent (Fig. 8(a)). In other words, forbidden patterns of *π*_3_ and *π*_6_ are observed if all oscillators are synchronized. The entropy ${ {\mathcal H} }_{O}$ shows parabola-like trends (increasing first and then decreasing slowly), but ${ {\mathcal H} }_{T}$ is a constant of 2.585 (Fig. 8(b)). All mean rotation frequencies converge to the same value since three oscillators are phase locked (Fig. 8(c)).

**Figure 9:**
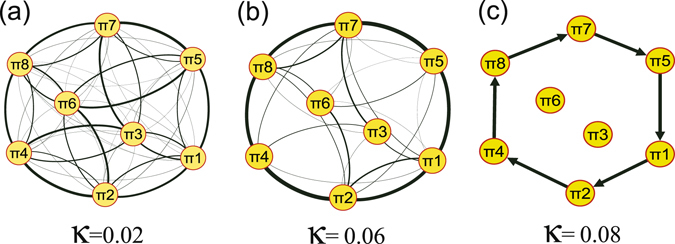
Ordinal transition networks on the path to phase synchronization of Eq. (8), for three typical coupling strength. (**a**) Random transitions in the non-sync regime of *κ* = 0.02 < *κ*_*c*1_, (**b**) dominant structure appears in the regime that oscillators *k* = 1 and *k* = 2 are phase synchronized, but not with *k* = 3, *κ* = 0.06 ∈ [*κ*_*c*1_, *κ*_*c*2_], (**c**) only one transition route of ordinal patterns is observed when all three oscillators are phase locked *κ* = 0.08 > *κ*_*c*2_. Thickness of links are determined by the associated frequencies in the transition networks and self-loops are removed. In (**a**,**b**), link arrows are suppressed for the ease of visualization.

From the viewpoint of high dimensional systems of coupled oscillators, in the process from non-synchrony to phase synchronization we find that the transition networks have experienced rather random transitions between all possible ordinal patterns to a state of transitions between a limited number of ordinal patterns as shown in Fig. 9. In addition, we find *π*_3_ and *π*_6_ are forbidden patterns if all three oscillators are synchronized.

## Conclusions

In this work, we have proposed to construct ordinal partition transition networks from multivariate time series, which help us to analyzing the interaction patterns between different components. The basic idea is to capture the directions of changes in the associated velocity space, which yields dynamic instead of static information in the original phase space. The resulting ordinal partition transition networks are weighted directed networks, which are fundamentally different to recurrence networks^19^ and visibility graphs^10^. For time series from both discrete and continuous dynamical systems, we find that the frequencies of observed ordinal patterns are inhomogeneous which is quantified by the entropy ${ {\mathcal H} }_{O}$. In addition, the transition frequencies between different ordinal patterns are inhomogeneous as well, which is characterized by the entropy ${ {\mathcal H} }_{T}$. Note that no essential difference between ${ {\mathcal H} }_{O}$ and ${ {\mathcal H} }_{T}$ is expected for discrete systems, however for continuous systems, ${ {\mathcal H} }_{T}$ is a better way to characterize the ordinal partition transition networks because ${ {\mathcal H} }_{O}$ is more influenced by self-loops as shown in Fig. 4.

The ordinal partition transition network utilizes nullclines to generate partitions, resulting in a Markov chain representation of the time series in phase space. The transition between two ordinal patterns is determined by the changes of signs of the increments of the variables. As we have demonstrated in the chaotic Rössler system, this definition is sensitive to capture the geometric changes in phase space, for instance, from phase coherence to non-coherence transition.

Note that our ordinal partition transition network generation algorithm is different to the recent work on constructing temporal networks to capture the memory effects^50^. It will be a future subject to characterize the memory effects by means of ordinal partition transition networks. In addition, we have focused on embedding dimension *D*_*x*_ = 2 and delay *τ* = 1 for each variable which captures either increasing or decreasing trends of a time series in differenced space. One can certainly generalize the algorithm to higher values of *D*_*x*_ and *τ*, however, it is computationally more demanding. For instance in a *n*-dimensional multivariate series $[\{{x}_{1}\}(t),\ldots ,\{{x}_{n}\}(t)]$, there are (3!)^*n*^ ordinal patterns if ${D}_{\{{x}_{1}\}}=\cdots ={D}_{\{{x}_{n}\}}=3$ is used. Additionally, the dimension of the transition matrix *W* is (3!)^*n*^ × (3!)^*n*^. Meanwhile, the increase of dimension *D*_*x*_ requires longer time series in order to estimate the transition frequencies of ordinal patterns more reliably. From the view point of the algorithm, no computational complexity is introduced by using a large time delay *τ* > 1, which, however, lacks a proper interpretation in terms of velocity of the variable. There is one open problem to estimate the ordinal partition transition matrix reliably from short time series, especially when noise plays a significant role.

We have applied ordinal partition transition networks to investigate the paths to phase synchronization, showing that a dominant transition route emerges in the networks when the coupling strength is increased undergoing different synchronization transition regimes. As the degree of synchronization is strengthened, dynamics of the coupled systems are locked to the synchronization manifold which yield a dominant transition route in the resulting ordinal partition networks. Before the appearance of synchronization, another challenging task is to distinguish the indirect from direct coupling directions^49, 51^, which is very common in climate data analysis, i.e., extracting network interaction patterns from multi-channel time series from distant places^52^. In the case of three coupled Rössler subsystems as we considered (Eq. (8)), the oscillator *k* = 2 is bidirectionally coupled to both *k* = 1 and *k* = 3, whereas there is no direct coupling between *k* = 1 and *k* = 3. It is possible to introduce ordinal recurrence plots^45^ to tackle this problem.

Traditionally, the computation of permutation entropy based on ordinal symbolic representation of time series does not include pattern transition behavior following the trajectory in phase space. In contrast, ordinal partition transition network approaches take into account the time evolution information explicitly, which hence provide much complementary insights to the traditional symbolic analysis, showing high potentials for experimental time series analysis.

## Methods: Phase coherence index

Here we summarize the major steps in computing phase coherence index as we have done in ref. 48. We restrict our attention in this work to the standard analytical signal approach. Here, a scalar signal *x*(*t*) is extended to the complex plane using the Hilbert transform

9 $$y(t)=\frac{1}{\pi }{\mathcal{P}}.{\mathcal{V}}.{\int }_{-\infty }^{\infty }\,\frac{x(t)-\langle x\rangle }{t-s}\,ds,$$

where ${\mathcal{P}}.{\mathcal{V}}.$ denotes Cauchy’s principal value of the integral, which yields the phase

10 $$\varphi (t)=\arctan \frac{y(t)}{x(t)}.$$

The above definition is straightforward for phase coherent dynamics. In the regime of non-phase coherent dynamics, an alternative phase definition has been proposed based on the local curvature properties of the analytical signal^53^, i.e.,

11 $$\tilde{\varphi }(t)=\arctan \frac{dy(t)/dt}{dx(t)/dt}.$$

Since in the standard Hilbert transform-based definition, the phase variable *ϕ*(*t*) does not necessarily increase monotonously in time, we quantify this monotonicity in order to obtain a simple heuristic order parameter for phase coherence, which we will refer to as the *coherence index*

12 $$CI=\mathop{\mathrm{lim}}\limits_{T\to \infty }\,\frac{1}{T}\,{\int }_{0}^{\infty }\,{\rm{\Theta }}(-\dot{\varphi }(t))\,dt$$

with $\dot{\varphi }(t)=d\varphi (t)/dt$. Furthermore, the instantaneous frequency of a chaotic oscillator is then defined as the derivative of the phase variable with respect to time. Averaging this property over time yields the mean frequency

13 $${\rm{\Omega }}=\frac{1}{2\pi }\langle \frac{d\varphi (t)}{dt}\rangle .$$
